# MicroRNA-enriched small extracellular vesicles possess odonto-immunomodulatory properties for modulating the immune response of macrophages and promoting odontogenesis

**DOI:** 10.1186/s13287-020-02039-1

**Published:** 2020-11-30

**Authors:** Jianmao Zheng, Yuanyuan Kong, Xiaoli Hu, Zhishan Li, Yaoyin Li, Yingqun Zhong, Xi Wei, Junqi Ling

**Affiliations:** 1 Guangdong Provincial Key Laboratory of Stomatology, Sun Yat-sen University, Guangzhou, Guangdong China; 2 Department of Operative Dentistry and Endodontics, Guanghua School of Stomatology, Affiliated Stomatological Hospital, Sun Yat-sen University, Guangzhou, 510055 Guangdong China; 3 Key Laboratory of Oral Medicine, Guangzhou Institute of Oral Disease, Department of Endodontics, Stomatology Hospital of Guangzhou Medical University, Guangzhou, Guangdong China

**Keywords:** Odonto-immunomodulation, Small extracellular vesicles, MicroRNAs, Odontogenesis, Macrophages, Dental pulp stem cells

## Abstract

**Background:**

To investigate the odonto-immunomodulatory properties of dental pulp stem cell-derived small extracellular vesicles (DPSCs-sEV), which promote odontogenesis by switching macrophages toward the pro-healing M2 phenotype.

**Methods:**

MicroRNA sequencing was carried out for microRNA profiling of DPSCs-sEV. Automated Western blot, qPCR, ELISA, and flow cytometry were performed to identify the functions of microRNA-enriched DPSCs-sEV in macrophages. A luciferase reporter gene assay was carried out to confirm exosomal miR-125a-3p’s direct target gene. DPSCs-sEV-stimulated macrophage-conditioned media were used to promote odontogenesis in DPSCs and explore the mechanism of immune response in DPSCs-SEV-stimulated odontogenesis. DPSCs-sEV were injected into the exposed pulp tissue of rat incisor to investigate the odonto-immunomodulatory properties of DPSCs-sEV in vivo.

**Results:**

DPSCs-sEV switched macrophages to the pro-healing M2 phenotype by inhibiting TLR and NFκΒ signaling. MicroRNA sequencing found 81 microRNAs significantly altered in DPSCS-sEV, with miR-125a-3p showing a 12-fold upregulation. Exosomal miR-125a-3p switched macrophages toward the M2 phenotype via inhibiting NFκΒ and TLR signaling via direct IKBKB targeting. Interestingly, DPSCs-sEV and the encapsulated miR-125a-3p enhanced BMP2 release in macrophages, promoting odontogenesis in DPSCs through BMP2 pathway activation. The rat study confirmed that DPSCs-sEV could be used as ideal biomimetic tools to enhance odontogenesis by switching macrophages toward pro-healing M2 cells.

**Conclusions:**

We firstly defined the odonto-immunomodulatory properties of microRNA-enriched DPSCs-sEV, which could be used as ideal biomimetic tools to enhance odontogenesis by switching macrophages toward the pro-healing M2 phenotype.

## Introduction

Maintaining a vital dental pulp has extremely high importance in dentistry [1] and is critical in developing favorable biomaterials facilitating the repair of the injured pulp [2]. However, as exogenous bodies, biomaterials could elicit significant immune reactions, which may play a pivotal role in dental pulp healing and the repair process [3]. Initially, inflammatory cytokines enhance generative dentin formation by odontoblasts, as well as dental pulp stem cell (DPSC) differentiation [4, 5]. However, persistent production of inflammatory cytokines would sustain inflammatory reactions in the pulp, ultimately leading to tissue necrosis [5, 6]. The above findings suggest that pulp capping biomaterials would be capable of immunomodulation, regulating immune reactions to promote pulp healing.

Of all immune cells in dental pulp, macrophages constitute important effector cells and play a pivotal role in the innate immune response [5]. Activated macrophages can be divided into classically (M1) and alternatively (M2) activated cells. M1 macrophages exert pro-inflammatory functions through helper T (Th) type 1 (Th1)-associated immune reactions [7], whereas M2 macrophages enhance Th2-related reactions, downregulating inflammation [8]. Besides regulating immune reactions, macrophages affect the odontogenic differentiation of stem cells as well as dental pulp repair [9, 10]. Macrophages can release regulatory factors such as BMP2, TGFβ1, and TGFβ3 [11–13], to actively participate in odontogenic differentiation of DPSCs [12]. During pulp tissue bioengineering, macrophages in the repaired pulp tissue phenotypically switch from M1 to M2, creating an optimal microenvironment for pulp tissue repair [9, 10].

DPSCs have immunomodulatory properties to modulate the function of macrophages [14]. Small extracellular vesicles derived from DPSCs (DPSCs-sEV) are considered to play a major role in the communication between DPSCs and target cells [15]. Thus, we speculated that the immunomodulatory effects of DPSCs are largely mediated by sEV. Recently, studies also suggested that DPSCs-sEV are ideal biomimetic tools for dental pulp regeneration by promoting odontogenesis in DPSCs [13, 16]. However, the effects of DPSCs-sEV on immune reactions associated with macrophages remain undefined; conversely, macrophages’ impact on DPSCs-sEV-regulated odontogenesis is unknown. Therefore, this study aimed to assess the interactions of biomimetic materials with macrophages, determining whether macrophages contribute to DPSCs-sEV-induced odontogenesis. Interestingly, we found DPSCs-sEV significantly modulated the immune response of macrophages, and shifted the immune microenvironment toward one that enhances odontogenesis. Thus, we firstly defined the odonto-immunomodulatory properties of DPSCs-sEV. This study provides novel insights into biomaterial-induced odontogenesis and an approach for optimizing systems to assess the odontogenic capability of dental biomaterials.

## Materials and methods

### Cell culture

This experiment has been approved to use third molars with indications for extraction by the Ethics Committee of Sun Yat-Sen University. Human DPSCs were harvested from healthy pulp tissues isolated from caries-free teeth of patients (5 females, age 24~35 years; 5 males, age 22~36 years) undergoing extraction of fully erupted third molars as reported [13]. Briefly, pulp tissue samples underwent digestion for DPSC isolation. The cells were cultured in DMEM containing 10% FBS (GIBCO, USA), 10 mg/ml streptomycin, and 10 U/ml penicillin (Sigma, USA) at 37 °C with 5% CO_2_. Passage 3–7 DPSCs were assessed. Macrophages RAW 264.7 cells as a gift from Dr. Zetao Chen (Guangdong Provincial Key Laboratory of Stomatology, Sun Yat-sen University, China) were cultured in the same condition.

### Isolation and identification of DPSCs-sEV

DPSCs-sEV were obtained from serum-free DMEM containing DPSCs as reported previously [13]. The markers CD9 and CD63 (Affinity, USA) were measured by automated Western blot analysis. Nano-flow cytometry (Nanofcm, UK) was performed to determine the diameter of DPSCs-sEV. DPSCs-sEV were detected and morphologically characterized on an H-7650 transmission electron microscope (HITACHI, Japan).

### Fluorescent labeling of DPSCs-sEV uptake by macrophages

Endocytosis of DPSCs-sEV by macrophages was measured by fluorescent labeling as reported previously [13]. PKH26-labeled DPSCs-sEV underwent incubation in the presence of macrophages for 24 h. Then, a confocal laser scanning microscope (Zeiss, Germany) was employed for detecting DPSCs-sEV endocytosis by macrophages. We randomly selected four fields to analyze PKH26-positive macrophages.

### Flow cytometry analysis

Phenotype markers of macrophages were determined by flow cytometry analysis.

For M1 macrophage marker CD11c, 100 μl macrophage suspension at 10^6^ cells/ml was added to 5 μl anti-CD11c-FITC (BioLegend, USA), followed by incubation at 37 °C for 30 min, centrifugation, two PBS washes, and flow cytometry analysis (BD, USA). Negative control staining was performed using the corresponding isotype control antibodies.

For M2 macrophage marker CD206, it was tested by intracellular immunofluorescent staining. Briefly, cells were fixed in 1 ml/tube Fixation Buffer (Beyotime, China) in the dark for 15 min at room temperature. Then, cells were permeabilized in 1 ml/tube Permeabilization Buffer Triton X-100 (Beyotime, China) for 10 min at room temperature. Finally, 100 μl fixed/permeabilized macrophage suspension at 10^6^ cells/ml was added to 5 μl anti-CD206-FITC (BioLegend, USA), followed by incubation at 37 °C for 30 min, centrifugation, two PBS washes, and flow cytometry analysis (BD, USA). Negative control staining was performed using the corresponding isotype control antibodies.

### Enzyme-linked immunosorbent assay

The amounts of cell-secreted inflammatory cytokines IL-10, IL-1ra, IL-1β, IL-6, and TNF-α were assessed with specific enzyme-linked immunosorbent assay (ELISA) kits (MEIMIAN, China) following the manufacturer’s instructions.

### Automated immunoblot

Automated immunoblot was carried out with Simple Wes (Protein Simple, USA) as described by the manufacturer. In brief, 1.5 μg total protein (cell lysate or exosome specimens) was loaded into a Wes assay plate, alongside antibody diluent (Protein Simple); primary antibodies targeting DSP (Santa Cruz, USA), DMP1, CD9, CD63, p-NFκΒ, IκB-a, Myd88, TLR4, IKBKB, BMP2, BMPR1, TGFβ1, TGFβ3, TGFR1, p-Smad2/3, p-Smad1/5/9, Smad4, β-actin, and β-Tublin (Affinity, USA); and anti-rabbit/mouse secondary antibodies (Protein Simple) and streptavidin-HRP. The compass software (Protein Simple) was employed for data analysis.

### Real-time PCR

Total RNA extraction from cells was carried out with RNA extraction kit (Qiagen, China). Next, 2 μg total RNA underwent reverse transcription with a reverse transcription polymerase chain reaction (RT-PCR) system (Promega). Then, qRT-PCR was carried out with SYBR-Green PCR kit (Qiagen, China) as directed by the manufacturer on a LightCycler 480 (Roche, USA). Three independent experiments were performed, and the ΔΔCt method was employed for analysis. The primers for IL-10, IL-1ra, TNF-a, IL-6, IL-1b, BMP2, TGFβ1, TGFβ3, and IKBKB are shown in Supplemental Table S1.

### MicroRNA sequencing and bioinformatics

MicroRNAs isolated from DPSCs-sEV were purified and sequenced on Illumina Hi-Seq 2000. Library construction and microRNA sequencing were carried out by RiboBio Ltd. (China). MicroRNAs with ≥ 2 fold change were further assessed. TargetScan, miRanda, Pita, and RNAhybrid were searched for genes targeted by differentially expressed miRNAs. Gene Ontology (GO; http://www.geneontology.org) and Kyoto Encyclopedia of Genes and Genomes (KEGG; http://www.genome.jp/kegg/pathway.html) pathway analyses were carried out for detecting molecular functions, biological processes, and pathways related to the predicted microRNAs’ target genes.

### Dual-luciferase reporter assay

A luciferase reporter gene assay was performed to determine whether IKBKB was directly targeted by miR-125a-3p. Luciferase reporter plasmids harboring wild-type (IKBKB-WT) and mutant (IKBKB-MUT) 3′UTRs of IKBKB, respectively, were constructed. The 3′UTR luciferase vector (150 ng) underwent co-transfection into cells with miR-125a-3p mimic and miR-125a-3p mimic-control, respectively, with Lipofectamine 2000 (Invitrogen) for 48 h. Luciferase activity was assessed with Dual-Luciferase Reporter Assay Kit (Beyotime Biotechnology, China) as directed by the manufacturer.

### Alizarin Red S staining

Alizarin Red S staining was employed for assessing calcium deposition and nodule formation. Destaining was carried out with 10% cetylpyridinium chloride monohydrate buffer, followed by optical density reading at 575 nm.

### Effects of microRNA-enriched DPSCs-sEV on the immune response of macrophages

Macrophages (RAW 264.7 cells) seeded in 6-well plates at 50,000/well were pretreated by lipopolysaccharide (1 μg/ml) for 2 h. Then, macrophages were incubated for 1 and 2 days with 30 μg/ml DPSCs-sEV, and a culture medium containing 10% exosome-free FBS served as control. The macrophages also underwent transfection (miR-27a-5p mimics or inhibitor) for 2 days as described above.

### Effects of DPSCs-sEV-stimulated macrophages on the odontogenesis of DPSCs

DPSCs were seeded into 6-well plates at 1 × 10^5^/well and incubated for 24 h. Then, the culture medium was removed and replaced with DPSCs-sEV-stimulated macrophage-conditioned medium for 14 days. DPSCs-sEV-conditioned media, macrophage-conditioned media, and pure culture media were used as controls.

### In vivo experiments of DPSCs-sEV in dental pulp capping

The animal study had approval from the Ethics Committee of Sun Yat-Sen University. Six 8-week-old SD rats were anesthetized by intraperitoneally injecting 5% chloral hydrate. The dental pulp of the upper left incisor was exposed, and coronal pulp tissue samples were obtained using No. 1/2 round bur and K-files. The exposed area underwent rinsing with 5% sodium hypochlorite and 3% hydrogen peroxide. After further rinsing with saline, 30 μg DPSCs-sEV in 20 μl PBS was injected into the exposed pulp tissue of each upper left incisor. The upper right incisor of the same animal was injected with 20 μl PBS served as control. Cavity sealing was carried out with MTA (Dentsply Sirona, USA).

Observations were made at 3 days after the operation. In the present study, we euthanized the rats by cervical dislocation. The relevant teeth were extracted and fixed with 3% formalin. Then, 10% sterile EDTA was used to demineralize the teeth at 4 °C, followed by paraffin embedding and sectioning at 5 μm for immunohistochemistry and immunofluorescence.

### Immunohistochemistry and immunofluorescence

For immunohistochemistry, slides underwent incubation with anti-CD68, anti-CD163, and anti-CD11c (Affinity, USA) primary antibodies at 4 °C overnight, followed by successive incubations with biotinylated goat anti-rat IgG (1:100, Invitrogen) for 1 h and avidin-biotin-peroxidase complex for 30 min at ambient. The DAB substrate kit (R&D Systems) was employed for development. Positively stained cells were imaged under an optical microscope (Leica) at × 40. ImageJ 1.37v (NIH, USA) was used to quantitate the density of stained cells (cell count per 150 × 200 μm^2^ area).

For immunofluorescence, specimens underwent incubation with primary antibodies raised against DSP (Santa Cruz) and DMP1 (Affinity), respectively, followed by treatment with Alexa Fluor 594-linked goat anti-rat IgG (1:100, Invitrogen) for 60 min. Counterstaining was performed with DAPI, and a fluorescence microscope (Leica) was employed for analysis. We performed a semi-quantitative analysis of the expression of DSP and DMP-1 by ImageJ.

### Statistical analysis

Experiments were repeated thrice, and data are mean ± SD. Independent samples *t* test was performed for comparing group pairs. One-way ANOVA was carried out for multiple group comparisons, with post hoc Bonferroni correction. Data were analyzed with the SPSS software package 16.0 (SPSS). *P* < 0.05 indicated statistical significance.

## Results

### Endocytosis of DPSCs-sEV by macrophages

**Fig. 1:**
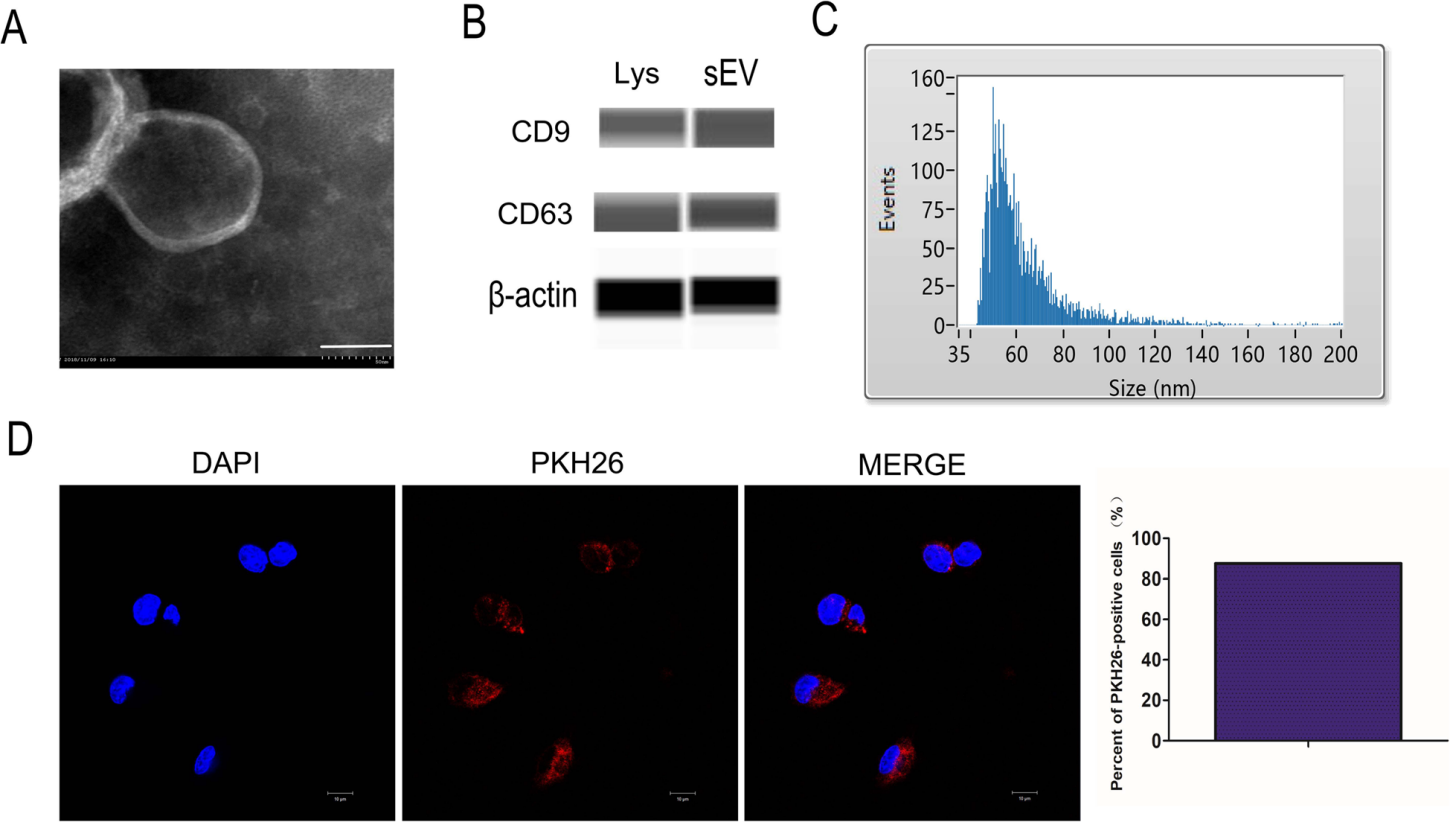
Identification and characterization of DPSCs-sEV. **a** The morphology of DPSCs-sEV was determined by TEM, scale bar = 50 nm. **b** Expression of CD9 and CD63 in the DPSCs-sEV (sEV represents DPSCs-sEV and Lys represents DPSCs lysate, β-actin is a control for the lysate). **c** Nano-flow cytometry showed DPSCs-sEV ranged between 30 and 150 nm in diameter. **d** PKH26-labeled DPSCs-sEV were found in macrophage cytosol

DPSCs-sEV were observed by TEM (Fig. 1a). Automated immunoblot demonstrated the expression of CD9 and CD63 in DPSCs-sEV (Fig. 1b). Nano-flow cytometry showed DPSCs-sEV ranged between 40 and 150 nm in diameter, the mean size of DPSCs-sEV was 61 nm, and the median size was 56 nm (Fig. 1c). To assess whether macrophages could endocytose DPSCs-sEV, PKH26 was employed for exosome labeling for 24 h. As shown in Fig. 1d, PKH26-labeled DPSCs-sEV were found in macrophage cytosol, and the percent of PKH26-positive cells were 87.5%.

### DPSCs-sEV’s effects on the immune response of macrophages

**Fig. 2:**
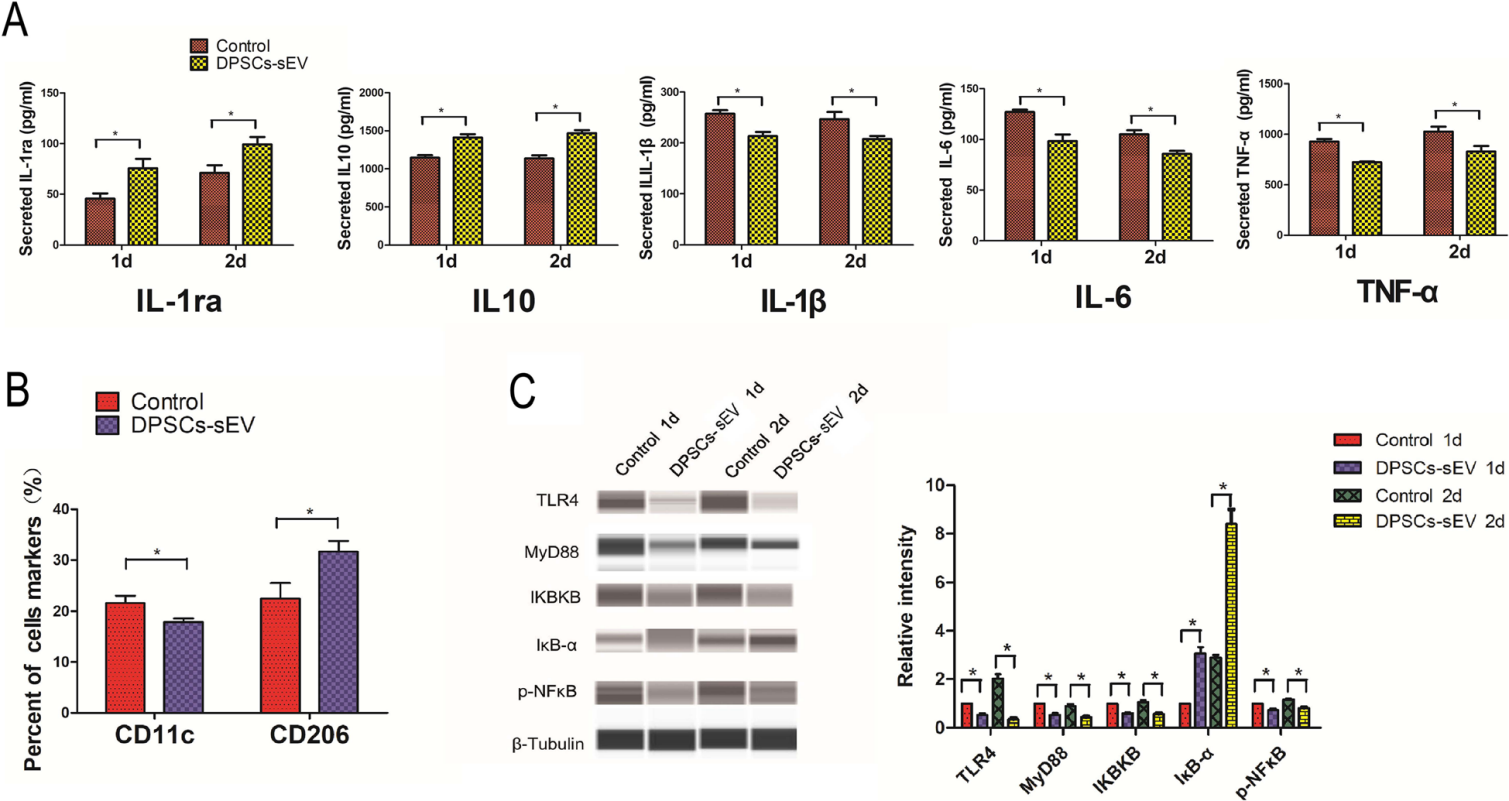
DPSCs-sEV manipulated the immune response of macrophages through NFκΒ signaling pathway. **a** DPSCs-sEV increased the secretion of anti-inflammatory cytokines IL-1ra and IL10, while decreased the pro-inflammatory cytokines IL-1β, IL-6, and TNF-α in macrophages. **b** When macrophages were treated with DPSCs-sEV for 2 days, the expression of M1 marker CD11c was inhibited, while the expression of M2 marker CD206 was enhanced. **c** When treated with DPSCs-sEV for 1 and 2 days, TLR4, MyD88, IKBKB, and p-NFκΒ were downregulated, while IκΒ-α was upregulating in macrophages

When macrophages were treated with DPSCs-sEV for 1 and 2 days, respectively, the secretion of IL-1ra and IL10 were significantly increased, while those of IL-1β, IL-6, and TNF-α were decreased (Fig. 2a). In addition, M1 (CD11c) and M2 (CD206) phenotypic markers were assessed, as shown in Fig. 2b and Supplemental Figure S3, and CD11c was suppressed, while CD206 was upregulated. These data implied that endocytosis of DPSCs-sEV resulted in macrophage polarization toward the pro-healing phenotype.

### DPSCs-sEV manipulate the immune response of macrophages through the TLR and NFκΒ signaling pathways

The results demonstrated that DPSCs-sEV inhibited TLR and NFκΒ signaling by downregulating TLR4, MyD88, IKBKB, and p-NFκΒ, and upregulating IκΒ-α in macrophages (Fig. 2c). These findings indicated that DPSCs-sEV dampened inflammatory reactions in macrophages likely via TLR and NFκΒ pathway suppression.

### DPSCs-sEV modulate immune reactivity via transfer of exosomal microRNAs

#### MicroRNA sequencing and bioinformatics of DPSCs-sEV

**Fig. 3:**
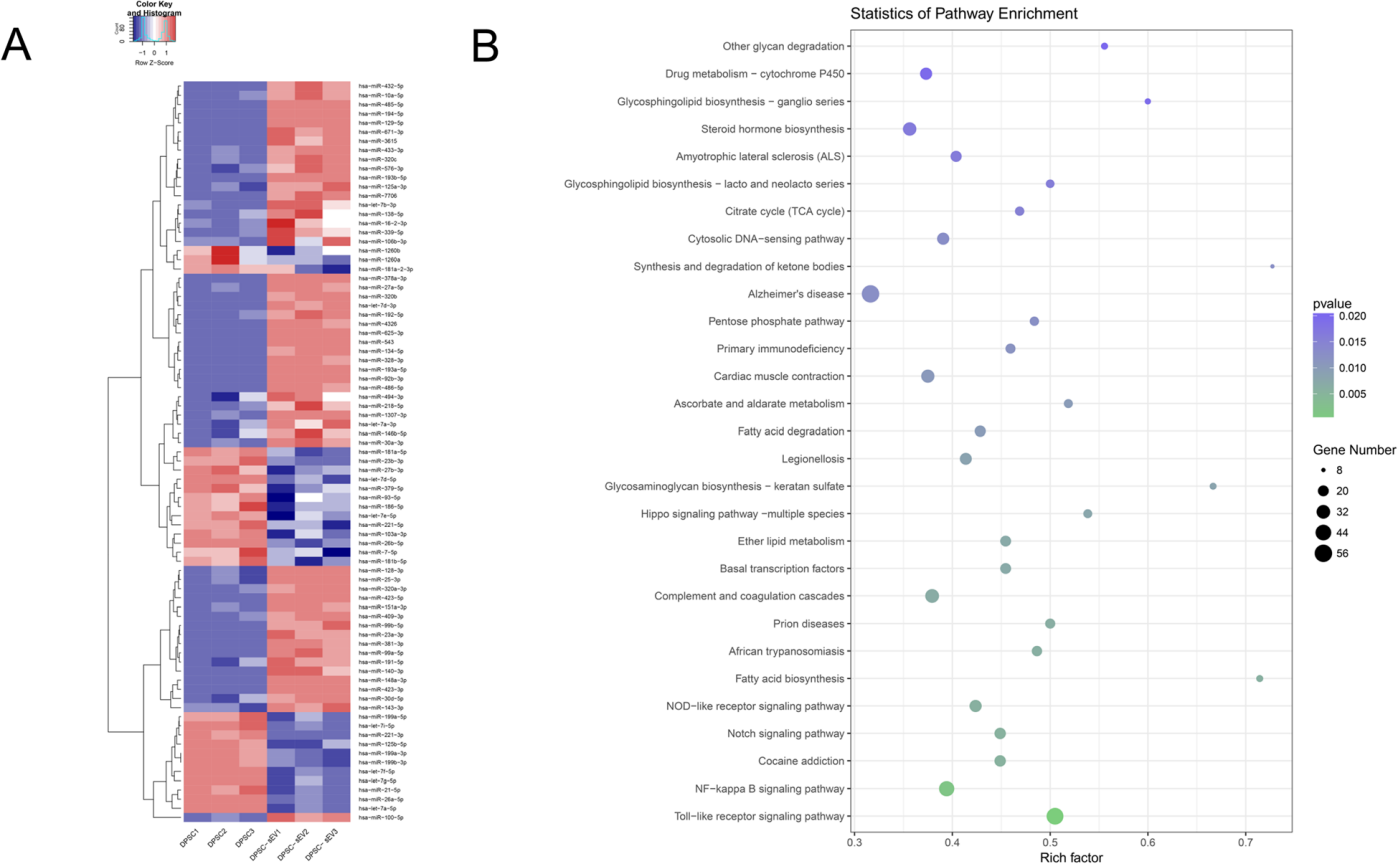
MicroRNA sequencing and bioinformatics analysis. **a** There were 81 microRNAs with a significant change in DPSCs-sEV, including 54 and 27 upregulated and downregulated, respectively. **b** Pathway analysis showed differentially expressed microRNAs’ targeted genes involved in many signal transduction pathways, such as NFκΒ and TLR signaling

We found that the amounts of microRNAs in DPSCs-sEV were markedly altered in comparison with their parent DPSCs. There were 81 microRNAs with a significant change in DPSCs-sEV, including 54 and 27 upregulated and downregulated, respectively (Fig. 3a, Supplemental Table S2). Next, TargetScan, miRanda, Pita, and RNAhybrid were employed for analyzing the gene targets of microRNAs with differential expression (Supplemental Figure S1). Pathway analysis revealed that these target genes participated in many signal transduction pathways, such as NFκΒ and TLR signaling (Fig. 3b), which were important in modulating the immune response of macrophages. Next, mRNA-microRNA interaction networks showed 41 genes in the NFκΒ pathway targeted by 53 microRNAs with differential expression (Supplemental Figure S5), and 51 in TLR signaling regulated by 56 microRNAs with differential expression (Supplemental Figure S6).

#### miR-125a-3p contained in DPSCs-sEV polarized macrophages toward M2 phenotype through NFκΒ signaling pathway by directly targeting IKBKB

**Fig. 4:**
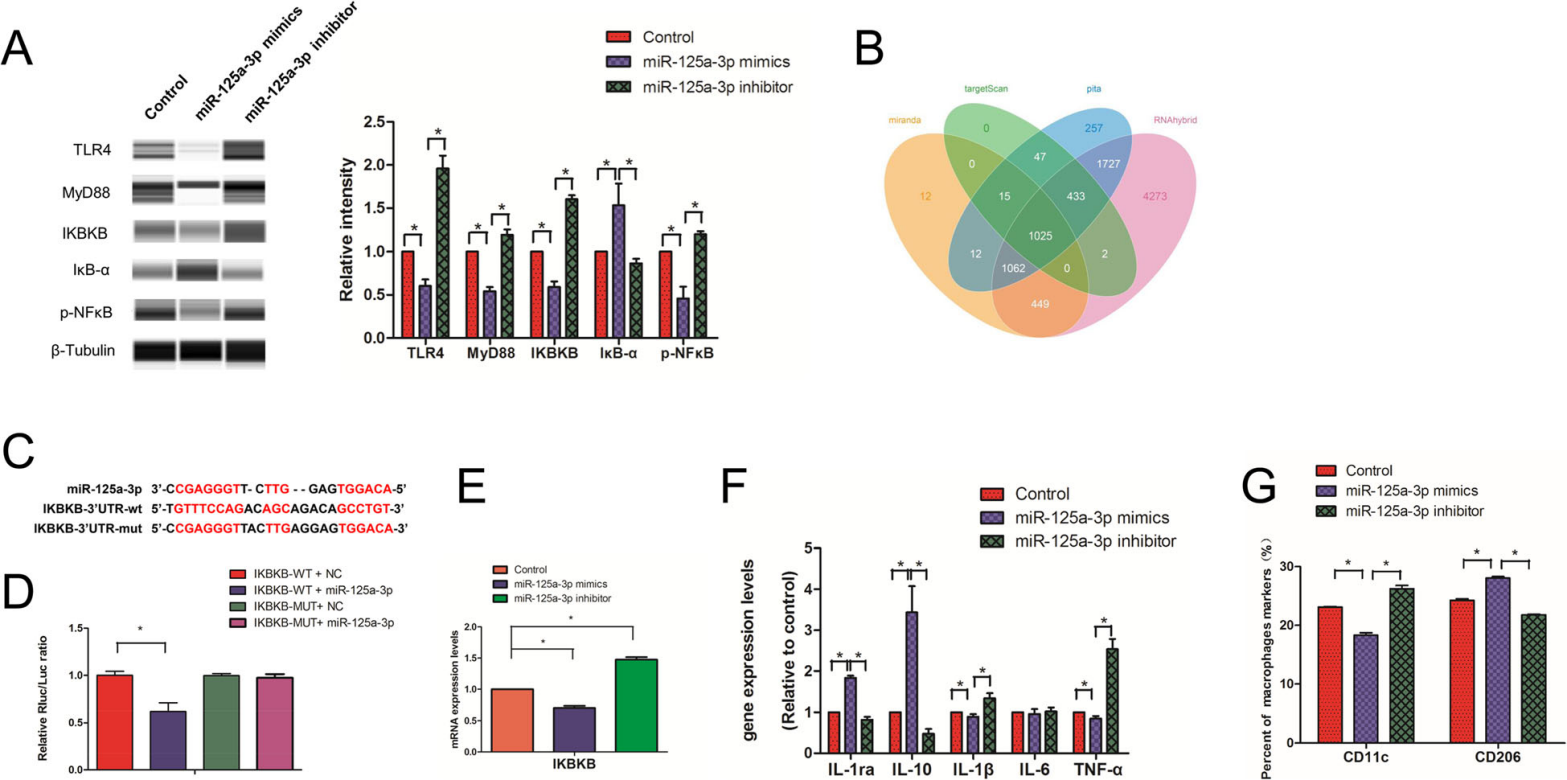
miR-125a-3p contained in DPSCs-sEV manipulated immune response of macropahges through NFκΒ and TLR pathway by directly targeting IKBKB. **a** miR-125a-3p downregulated TLR4, MyD88, IKBKB, and p-NFκΒ, but upregulating IκΒ-α in macrophages. **b** IKBKB was found to be targeted by miR-125a-3p by four bioinformatics tools. **c** The predicted miRNA binding sites in the 3′UTR of IKBKB by miR-125a-3p. **d** Double-luciferase assay and **e** qPCR determined that IKBKB could be directly downregulated by miR-125a-3p. **f** IL-1ra and IL10 were increased, whereas the IL-1β and TNF-α were decreased in macrophages treated with miR-125a-3p. **g** The expression of M1 markers was inhibited, whereas the expression of M2 markers was enhanced by miR-125a-3p

Among these microRNAs, miR-125a-3p showed a 12-fold upregulation in DPSCs-sEV (Supplemental Table S2) and was predicted to regulate the NFκΒ and TLR pathways (Supplemental Figures S5 and S6). Automated immunoblot demonstrated that miR-125a-3p mimics downregulated TLR4, MyD88, IKBKB, and p-NFκΒ, while upregulating IκΒ-α in macrophages (Fig. 4a). It is known that IKBKB inhibits IκΒ-α and activates the NFκΒ signaling pathway [17]. IKBKB was predicted as a miR-125a-3p target by the four bioinformatics tools (Fig. 4b), and the probable miRNA binding sites in IKBKB’s 3′UTR are depicted in Fig. 4c. We used qPCR and double-luciferase assays to determine that IKBKB was directly downregulated by miR-125a-3p (Fig. 4d, e), indicating that miR-125a-3p inhibited the NFκΒ signaling pathway by directly targeting IKBKB.

To confirm the effect of microRNAs contained in DPSCs-sEV on the immune response of macrophages, they were treated with miR-125a-3p mimics or inhibitor for 2 days, and IL-1ra and IL10 were significantly upregulated, while IL-1β and TNF-α showed decreased amounts after treatment with miR-125a-3p mimics (Fig. 4f). In agreement, CD11c and CD206 in macrophages were suppressed and upregulated by miR-125a-3p mimics, respectively (Fig. 4g and Supplemental Figure S4).

### Expression of odontogenic factors in macrophages induced by DPSCs-sEV and the encapsulated microRNAs

**Fig. 5:**
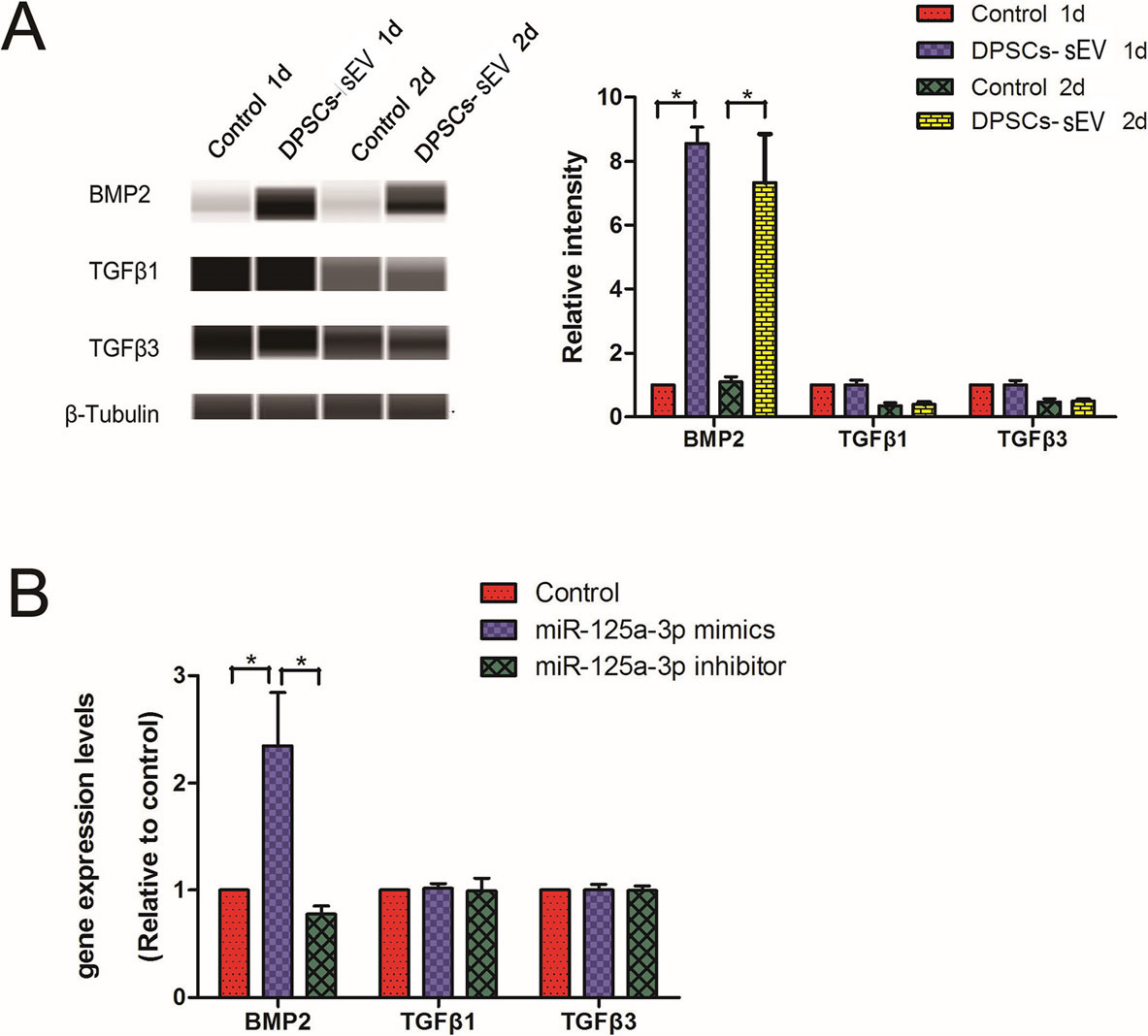
Odontogenic factor expression in macrophages induced by DPSCs-sEV and their encapsulated microRNAs. **a** DPSCs-sEV upregulated the protein expression of BMP2, but did not change the TGFβ1 and TGFβ3. **b** When macrophages were treated with miR-125a-3p for 2 days, the gene expression of BMP2 increased, but the TGFβ1 and TGFβ3 did not change

Macrophages can release odontogenic factors to actively participate in odontogenesis [12]. BMP2, TGFβ1, and TGFβ3 are involved in odontogenesis [11–13]. For full definition of the odonto-immune environment, such molecules should be evaluated. When macrophages were treated with DPSCs-sEV for 1 and 2 days, respectively, BMP2 protein amounts were markedly increased compared with the control group, whereas both TGFβ1 and TGFβ3 amounts showed no changes (Fig. 5a). We also determined the impact of exosomal miR-125a-3p on the expression of odontogenesis-related genes. Exosomal miR-125a-3p increased BMP2 mRNA amounts, but did not change TGFβ1 and TGFβ3 levels in macrophages after 2 days (Fig. 5b). These results indicated that macrophages treated with DPSCs-sEV provided a suitable odonto-immune environment for odontogenesis.

### DPSCs-sEV-stimulated macrophages provide a suitable odonto-immune environment for odontogenesis of DPSCs through BMP signaling

**Fig. 6:**
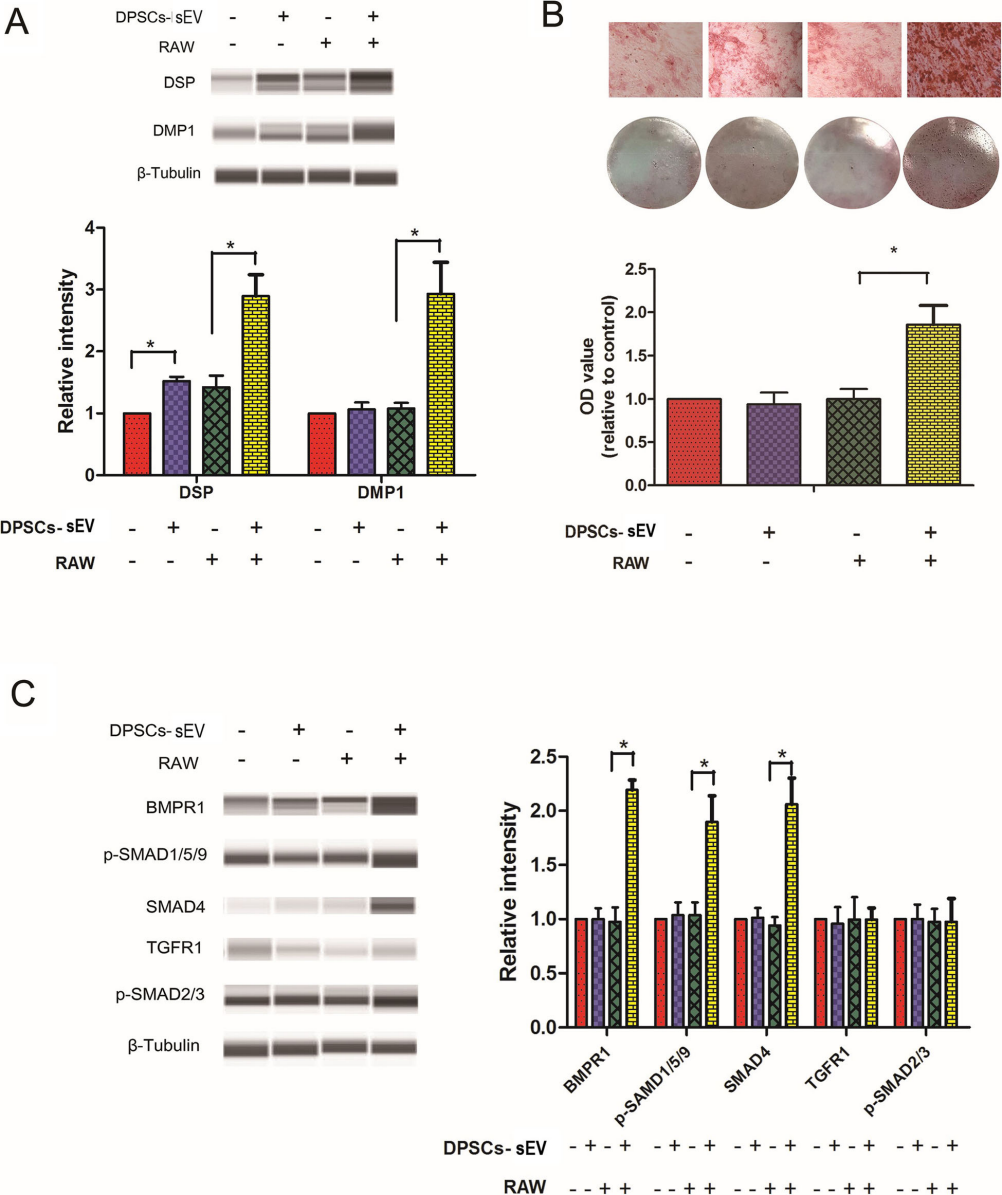
DPSCs-sEV-stimulated macrophage-conditioned medium enhances odontogenic differentiation in DPSCs via BMP signaling. **a** DPSCs-sEV-stimulated macrophage-conditioned medium upregulated DSP and DMP-1 proteins and **b** enhanced calcium deposition and nodule formation in DPSCs. **c** DPSCs-sEV-stimulated macrophage-conditioned medium increased the protein expression amounts of BMPR1, p-SMAD1/5/9, and SMAD4 in DPSCs, without altering TGFR1 and p-SMAD2/3 levels

After treatment with DPSCs-sEV-stimulated macrophage-conditioned media for 14 days, DPSCs had significantly upregulated DSP and DMP1 proteins (odontoblast-specific markers), compared with the control group (Fig. 6a). More calcium accumulation and nodule formation were found in DPSCs incubated with DPSCs-sEV-stimulated macrophage-conditioned medium (Fig. 6b). This indicated that DPSCs-sEV-stimulated macrophages provided a suitable odonto-immune environment for odontogenic differentiation in DPSCs.

To explore the mechanisms of enhanced odontogenesis in DPSCs treated with DPSCs-sEV-stimulated macrophage-conditioned medium, proteins involved in BMP and TGFβ signaling were assessed. As shown in Fig. 6c, BMPR1, p-SMAD1/5/9, and SMAD4 protein amounts in DPSCs were significantly elevated, whereas those of TGFR1 and p-SMAD2/3 remained unchanged. These data indicated BMP pathway activation in DPSCs under induction by DPSCs-sEV-stimulated macrophage-conditioned media, while the TGFβ signaling pathway was inactivated.

### DPSCs-sEV switch macrophages toward the pro-healing M2 phenotype promoting odontogenesis in vivo

**Fig. 7:**
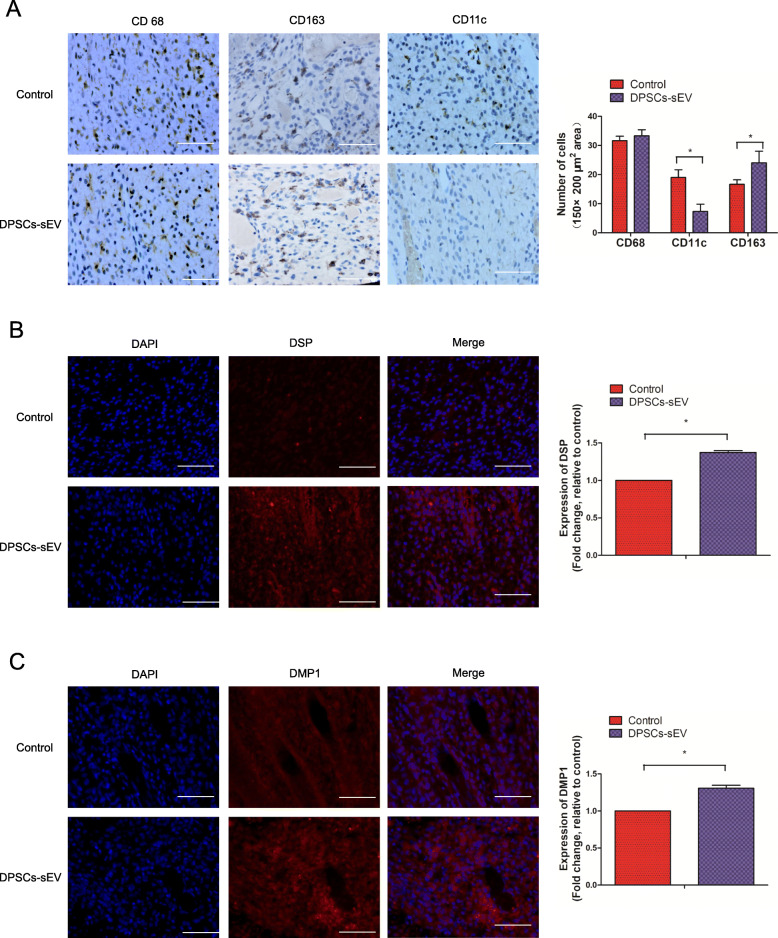
DPSCs-sEV polarize macrophages toward the pro-healing M2 phenotype that promotes odontogenesis in vivo. **a** The amounts of CD163-immunopositive M2 macrophages were remarkably increased in dental pulp tissue treated with DPSCs-sEV, while fewer CD11c-immunopositive M1 macrophages were observed, compared with the control group. The odontoblast-specific markers DSP (**b**) and DMP-1 (**c**) were significantly upregulated in dental pulp tissue incubated with DPSCs-sEV, compared with the control group

To investigate whether DPSCs-sEV modulate the immune response of macrophages to promote odontogenesis in vivo, DPSCs-sEV used as a pulp capping biomaterial was injected into the exposed pulp tissue of rat incisor. The results showed that CD163-immunopositive M2 macrophages were markedly more abundant in dental pulp tissue treated with DPSCs-sEV, while fewer CD11c-immunopositive M1 macrophages were observed, compared with the control group (Fig. 7a). Consistent with results of in vitro experiments, we found significantly upregulated DSP and DMP-1 proteins (odontoblast-specific markers) in dental pulp tissue treated with DPSCs-sEV compared with control specimens (Fig. 7b, c). These results indicated that DPSCs-sEV could switch macrophages toward the pro-healing M2 phenotype, which promoted odontogenesis in vivo.

## Discussion

**Fig. 8:**
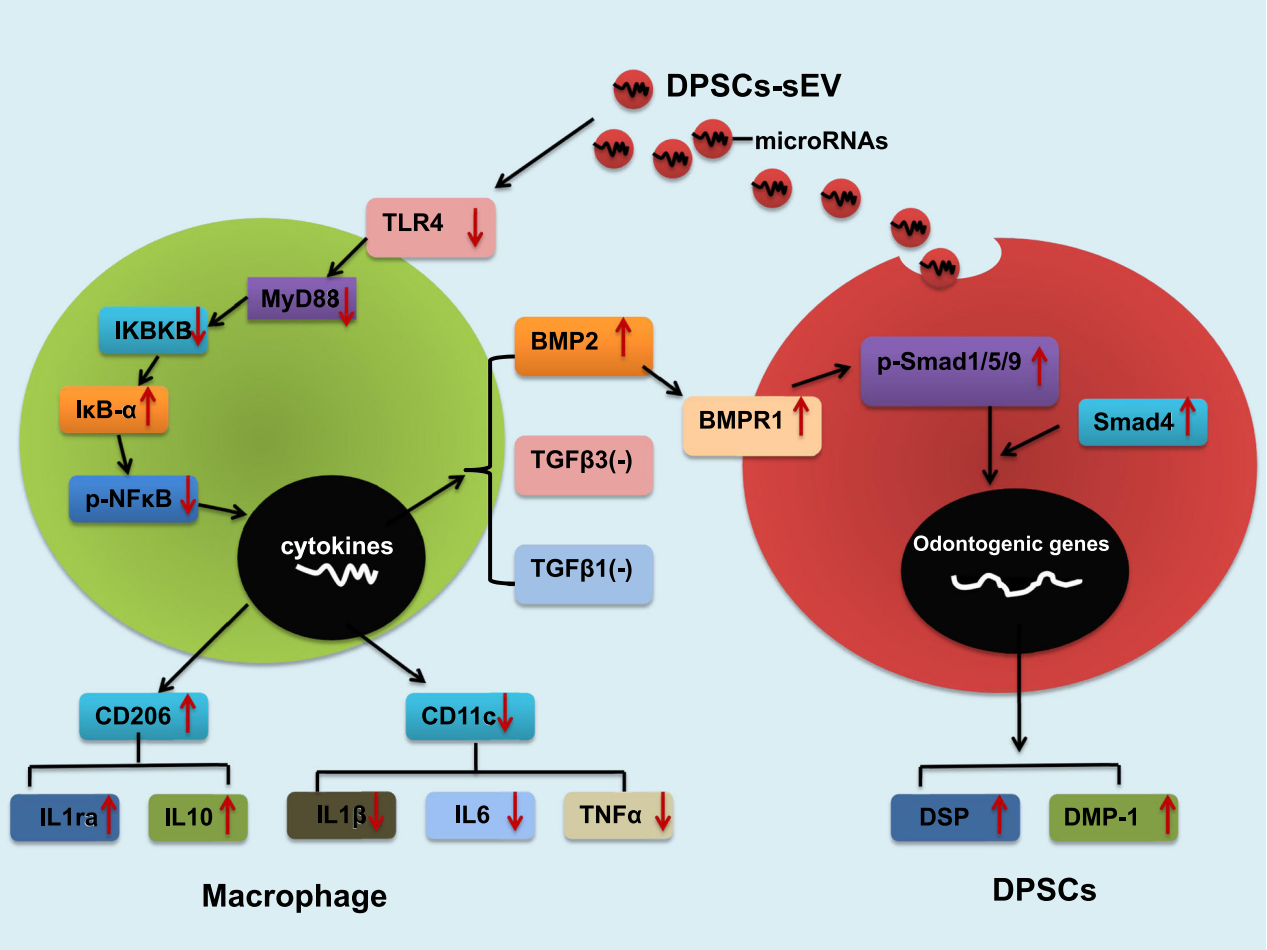
Schematic diagram depicting the detailed mechanisms of DPSCs-sEV regulating the immune response of macrophages in odontogenesis

The current work demonstrated that in response to DPSCs-sEV and the encapsulated microRNAs, macrophages could polarize to the M2 phenotype via TLR and NFκΒ pathway suppression, resulting in increased release of anti-inflammatory cytokines and the odontogenic factor BMP2, which promoted odontogenesis in DPSCs via BMP signaling (Fig. 8). These results demonstrated macrophages significantly contribute to DPSCs-sEV associated odontogenesis. Based on these findings, we firstly defined the odonto-immunomodulatory properties of DPSCs-sEV, which should be used as ideal biomimetic tools for pulp regeneration.

In the present study, we adopted the commercial precipitation kits to isolate the exosomes or sEV, and the results showed that we successfully isolated the DPSCs-sEV. As reported, EV also can be isolated and concentrated by differential ultracentrifugation [18]. Enrichment of different EVs is obtained at each step of centrifugation. EV larger than 200 nm (lEV) are pelleted at low-speed centrifugation (2000×*g*), but the EV smaller than 150 nm (sEV) recovered at the last step of high-speed ultracentrifugation (100,000×*g*) [19]. In the literature, the latter EVs are generally called exosomes [20]. However, we now know that this small EV pellet is composed not only of exosomes but also of other non-exosomal vesicles [19]; thus, we will use the term “small EV” instead of “exosomes” [21]. As reported, lEV and sEV derived from immature dendritic cells differ in their capacity to orient T helper (Th) cell responses, the former induce secretion of Th2 cytokines, whereas the latter promote Th1 cytokine secretion [22]. It reveals that different subtypes of EVs might have different biological functions. In the current study, we have confirmed the biological functions of DPSCs-sEV, but do lEV derived from DPSCs have the same effects on macrophages, or have different effects? Do different technical methods affect the size of the DPSCs-sEV isolated? Are the DPSCs-sEV obtained by ultracentrifugation and precipitation different? So far, it is still unclear and needs further research and clarification.

### Effects of DPSCs-sEV on macrophages

Macrophages constitute plastic and dynamic cells, whose phenotype switches between the M1 and M2 extremes, based on environmental conditions [23]. Whether macrophages respond to DPSCs-sEV by phenotype switching remains unclear. We found in this study that DPSCs-sEV uptake triggered more macrophages expressing the M2 marker CD206 and upregulated anti-inflammatory cytokines (IL-10 and IL-1ra). The above findings suggested that DPSCs-sEV exert effects on the polarization of macrophages, which switch toward a more anti-inflammatory phenotype.

Macrophages recognize antigens through TLR and NFκΒ signaling, inducing innate immunity for antigen degradation or expulsion [24]. TLR4 and MyD88 are essential effectors of TLR signaling; activated TLR4 interacts with MyD88, eventually activating the downstream NF-κB signaling, which upregulates pro-inflammatory cytokines [25]. IκΒ-α is an inhibitor of NFκB, and IKBKB downregulation and IκΒ-α upregulation inhibit the activity of NFκB, thereby impeding inflammation [17, 23]. In this work, TLR4, MyD88, IKBKB, and p-NFκΒ proteins were all downregulated in macrophages, while the NFκΒ inhibitor IκB-a was upregulated. These findings indicate that DPSCs-sEV regulate the inflammatory response of macrophages likely via TLR and NFκΒ pathway suppression.

The functions of microRNAs encapsulated in DPSCs-sEV on macrophages remain unrevealed. As shown above, 81 microRNAs had significant alterations in DPSCs-sEV, and their target genes participated in various signaling cascades such as NFκΒ and TLR pathways. Among these microRNAs, miR-125a-3p showed a 12-fold upregulation in DPSCs-sEV and was predicted to participate in NFκΒ and TLR signaling. Consistently, immunoblot showed that miR-125a-3p downregulated TLR4, MyD88, IKBKB, and p-NFκΒ proteins, while upregulating IκΒ-α in macrophages. IKBKB inhibits IκΒ-α and activates the NFκΒ signaling pathway [17]. We used double-luciferase assay to determine that IKBKB was directly downregulated by miR-125a-3p, indicating that miR-125a-3p contained in DPSCs-sEV inhibits TLR and NFκΒ signaling pathways by directly targeting IKBKB.

To confirm the effect of microRNAs contained in DPSCs-sEV on the immune response of macrophages, these cells were treated with miR-125a-3p. As a result, the M2 marker CD206 and anti-inflammatory cytokines were significantly enhanced, while the M1 marker CD11c and pro-inflammatory cytokines were significantly reduced. These data indicated that DPSCs-sEV switch macrophages toward a more pro-healing phenotype by transfer of microRNAs.

However, sEV contain a variety of substances, such as proteins and ncRNA (miRNA, lncRNA, circRNA, etc.). Studies have found that proteins and ncRNA contained in exosomes or sEV can all act on the M2 polarization of macrophages [26–28], but which one plays a key role is currently difficult to conclude. We think it depends on which cell or tissue the sEV originate from, and the macrophages of which organs or tissues the sEV specifically act on. In view of the role of exosomes or sEV derived from dental pulp stem cells in this study, our future experiments will study the role of the proteins contained in DPSCs-sEV and further explore whether proteins or ncRNA contained in DPSCs-sEV play a key role in regulating the polarization of macrophages.

### DPSCs-sEV-stimulated macrophages enhance odontogenic differentiation in DPSCs

Macrophages have essential modulatory functions in tissue regeneration [29]. Indeed, macrophage recruitment is enhanced in the early phase of pulp regeneration [30]. Paula-Silva et al. [4] found that TNF-α released from M1 macrophages promotes the odontogenic differentiation of dental pulp cells. However, macrophages undergo M1-to-M2 phenotypical switch in pulp tissue engineering, indicating that such macrophage transition is essential for building a suitable microenvironment for pulp tissue repair [9, 10]. M2 macrophages can release odontogenic factors, such as BMP2, TGFβ1, and TGFβ3, to actively participate in odontogenesis [11–13]. Therefore, effectively and adequately switching macrophage phenotype toward M2 might significantly enhance odontogenesis.

Macrophages can produce BMP2, which transmits signals via SMAD-dependent and SMAD-independent pathways, resulting in odontogenic differentiation of mesenchymal stem cells [8, 11]. As shown above, DPSCs-sEV significantly elevated the release of BMP2 in macrophages, which in turn activated the BMP signaling pathway by increasing BMPR1, p-SMAD1/5/9, and SMAD4 levels in DPSCs, upregulating the odontoblast-specific markers DSP and DMP-1, which results in odontogenesis of DPSCs. The mechanism of BMP2 upregulation in macrophages after DPSCs-sEV stimulation deserves further investigation. As reported previously, inhibition of NF-κB signaling could switch the macrophage phenotype to M2 and increase IL-10 release [8], which in turn could upregulate BMP2 by a positive feedback loop [31]. Consistent with previous findings, we determined that DPSCs-sEV and their encapsulated microRNAs switched macrophages to the M2 phenotype by inhibiting NFκΒ signaling, resulting in IL-10 upregulation, which in turn enhanced BMP2 expression.

Inflammatory cytokines initially enhance dentin synthesis by odontoblasts as well as DPSC odontogenesis [4, 5]. However, persistent expression of inflammatory cytokines would result in continuous inflammation in the pulp, ultimately leading to tissue necrosis [5, 6]. IL-1ra and IL-10 are anti-inflammatory molecules that suppress pro-inflammatory cytokine synthesis and may be involved in dentin formation [32–34]. We found that DPSCs-sEV switched macrophage phenotype from M1 to M2, which resulted in upregulated anti-inflammatory cytokines (IL-1RA and IL-10) and downregulated pro-inflammatory cytokines (IL-1β, IL-6, and TNF-α). These findings indicated that DPSCs-sEV-associated macrophage polarization does not induce inflammation, but adequately improves odontogenesis by upregulation of anti-inflammatory cytokines and BMP2, which activates BMP signaling in DPSCs, promoting odontogenic differentiation.

Finally, DPSCs-sEV was injected into the exposed pulp tissue of rat incisor, and the results showed that DPSCs-sEV upregulated the odontoblast-specific markers DSP and DMP-1 in dental pulp tissue by switching macrophages toward the pro-healing M2 phenotype, which promotes odontogenesis. Effective and adequate phenotype switching of macrophages from M1 to M2 represents a new strategy for pulp regeneration, which would be effective in creating a microenvironment favoring inflammation suppression, restoring the balance favoring tissue repair [5, 9, 10]. Parameters promoting such transition are essential while designing bioactive dental materials.

In the present study, we confirmed DPSCs-sEV-induced M2 macrophages could enhance odontogenic differentiation in DPSCs. But, what is the difference between DPSCs-sEV-stimulated M2 macrophages and M2 macrophages induced by other inducers, such as IL-4? Whether IL-4 induced M2 macrophages have the same effect on DPSCs? Meanwhile, it is still unknown and worthy of further exploration. A study performed by Jayme et al. [35] has shown that IL-4 can promote epithelial wound healing and reduce colitis by inducing the M2 polarization of macrophages. Other studies [9, 10, 12, 30] and our current research have shown that M2 macrophages can promote dental pulp repair and odontogenic differentiation in DPSCs. Therefore, we speculate that M2 macrophages induced by IL-4 or other inducing factors may promote the odontogenic differentiation of DPSCs and dental pulp repair, but this needs further research and verification.

## Conclusions

The present study revealed that microRNA-enriched DPSCs-sEV polarized macrophages to the pro-healing M2 phenotype by inhibiting the TLR and NFκΒ pathway. miR-125a-3p contained in DPSCs-sEV switched macrophages toward the M2 phenotype by inhibiting the NFκΒ and TLR pathway via direct targeting of IKBKB. Interestingly, DPSCs-sEV and the encapsulated miR-125a-3p enhanced BMP2 release by macrophages, which in turn promoted the odontogenesis of DPSCs through BMP2 pathway activation. The in vivo study confirmed that DPSCs-sEV polarized macrophages toward the pro-healing M2 phenotype to enhance odontogenesis. These findings identified the odonto-immunomodulatory properties of DPSCs-sEV, which could be used as ideal biomimetic tools to modulate the immune response of macrophages to induce odontogenesis.

## Supplementary Information

**Additional file 1: Fig. S1.** All genes targeted by differentially expressed microRNAs were shown

**Additional file 2: Figure S2.** Original non-edited images of Chemiluminescence intensity and the automatically generated bands for MyD88and β-tubulin. (A) DPSCs-sEV down-regulated the expression of MyD88 in macrophages. (B) miR-125a-3p down-regulated the expression of MyD88 in macrophages

**Additional file 3: Figure S3.** M1 (CD11c) and M2 (CD206) phenotypic markers were assessed

**Additional file 4: Figure S4.** CD11c and CD206 in macrophages were suppressed and upregulated by miR-125a-3p mimics

**Additional file 5: Figure S5.** mRNA-microRNA interaction networks showed 41 genes in the NFκΒ pathway targeted by 53 microRNAs with differential expression

**Additional file 6: Figure S6.** 51 in TLR signaling regulated by 56 microRNAs with differential expression

**Additional file 7: Table S1.** Primer pairs used in the qRT-PCR.

**Additional file 8 Table S2.** There were 81 microRNAs significantly changed in DPSCs-sEV, of which 54 increased and 27 decreased.

## Data Availability

The authors confirm the availability of all data generated or analyzed in this manuscript.

## Abbreviations

DPSCs: Dental pulp stem cells
DPSCs-sEV: Small extracellular vesicles derived from dental pulp stem cells
DSP: Dentin sialophosphoprotein
DMP-1: Dentin matrix protein 1
GO: Gene Ontology
BMP2: Bone morphogenetic protein 2
BMPR1: Bone morphogenetic protein receptor 1
NFκΒ: Nuclear factor kappa-B
TLR: Toll-like receptor
