# Supplementary figures and images for: MicroRNA-enriched small extracellular vesicles possess odonto-immunomodulatory properties for modulating the immune response of macrophages and promoting odontogenesis
Source: Stem Cell Res Ther. 2020 Nov 30;11:517. doi: 10.1186/s13287-020-02039-1 (PMC7708107; doi:10.1186/s13287-020-02039-1)

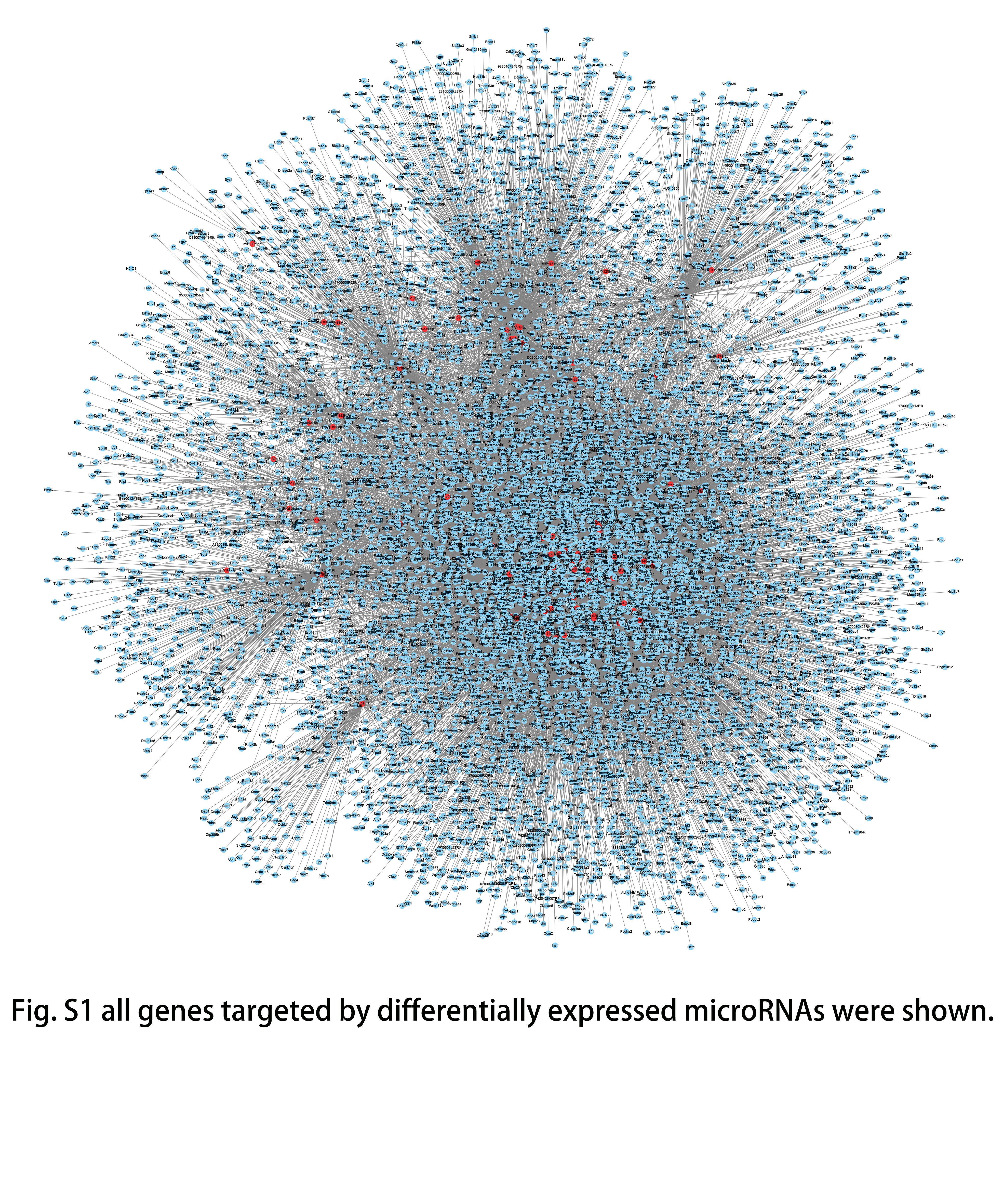

Supplement: Supplementary file 1 — Additional file 1: Fig. S1. All genes targeted by differentially expressed microRNAs were shown [file 13287_2020_2039_MOESM1_ESM.jpg]

# A

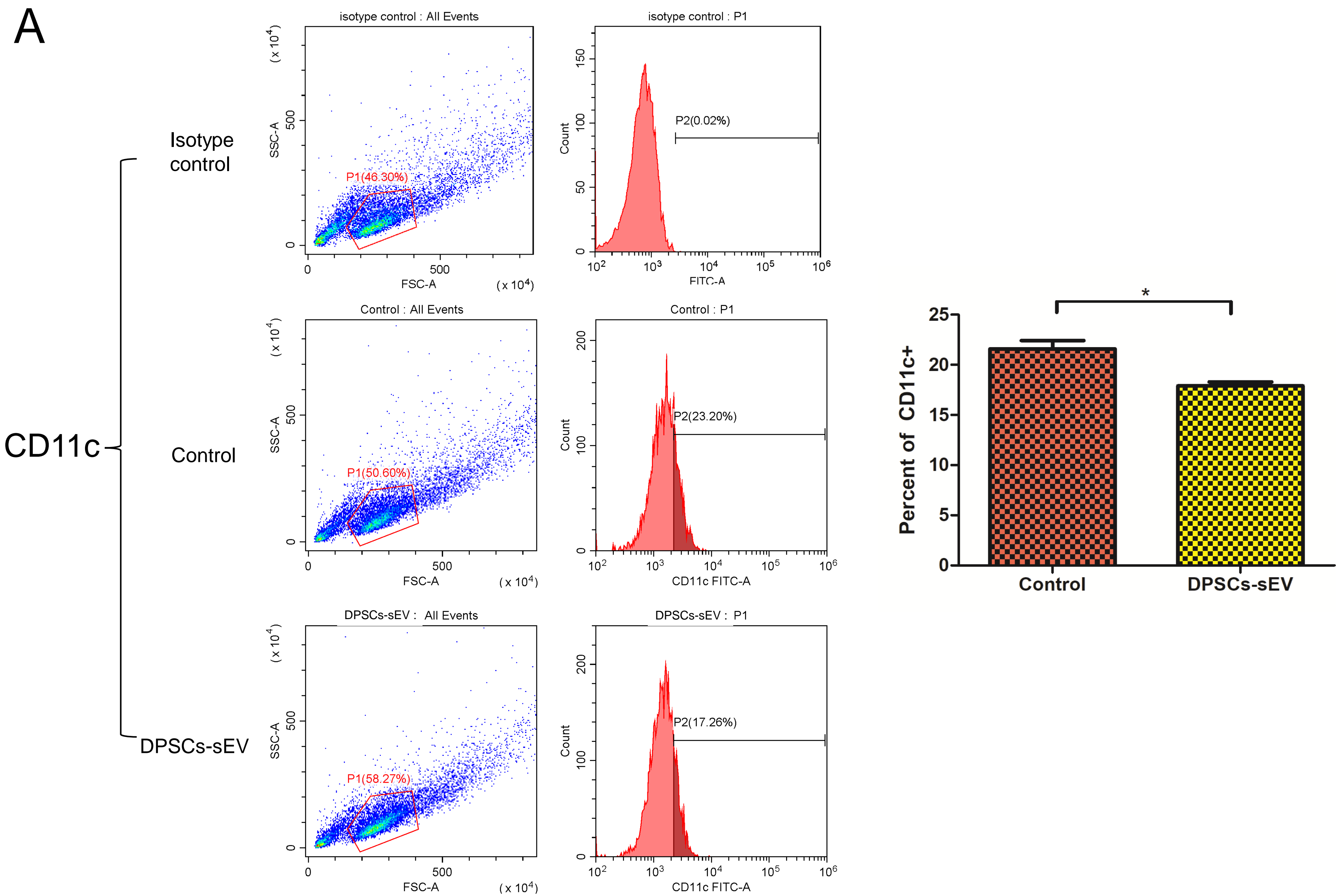

# B

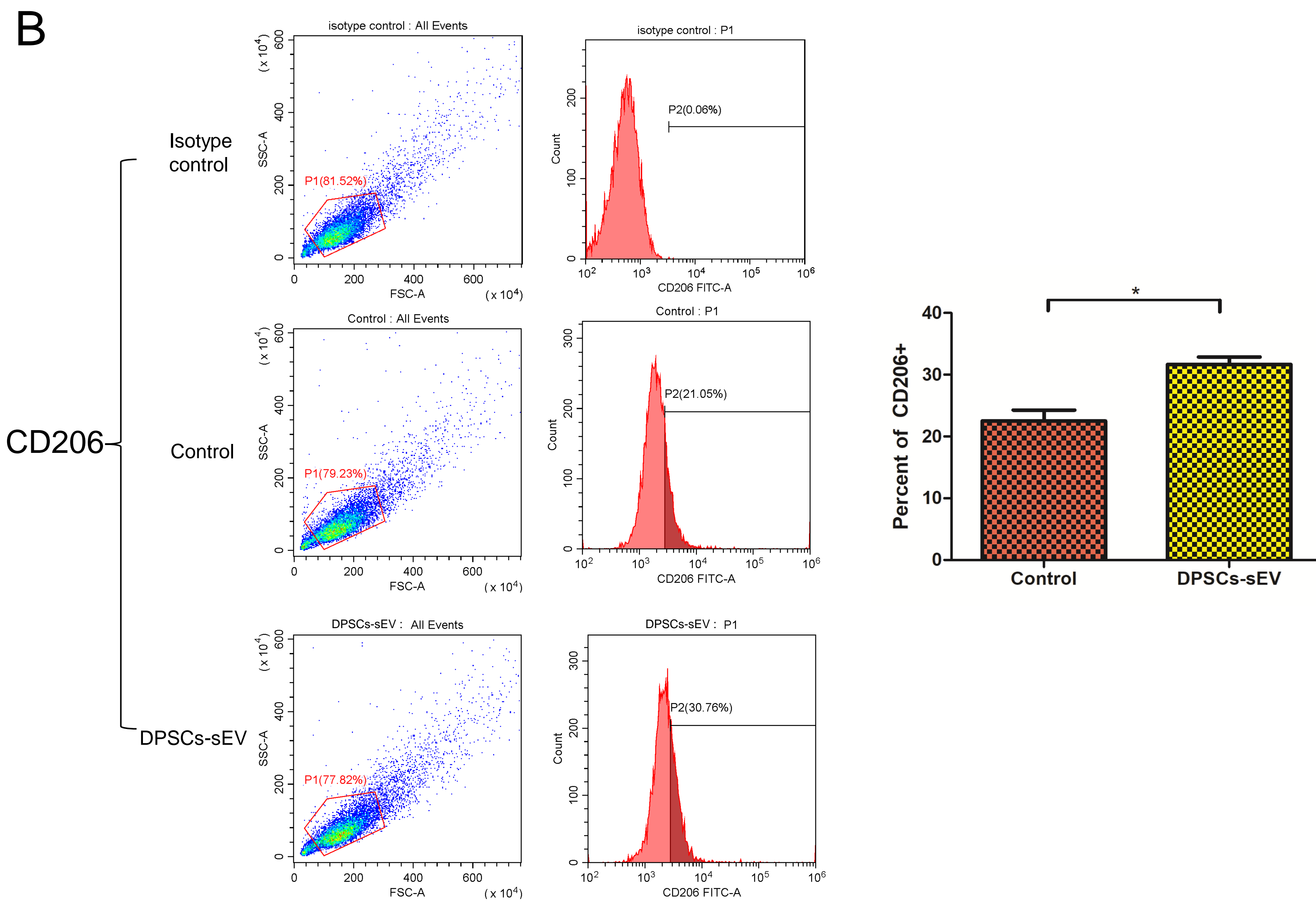

Supplement: Supplementary file 3 — Additional file 3: Figure S3. M1 (CD11c) and M2 (CD206) phenotypic markers were assessed [file 13287_2020_2039_MOESM3_ESM.pdf]

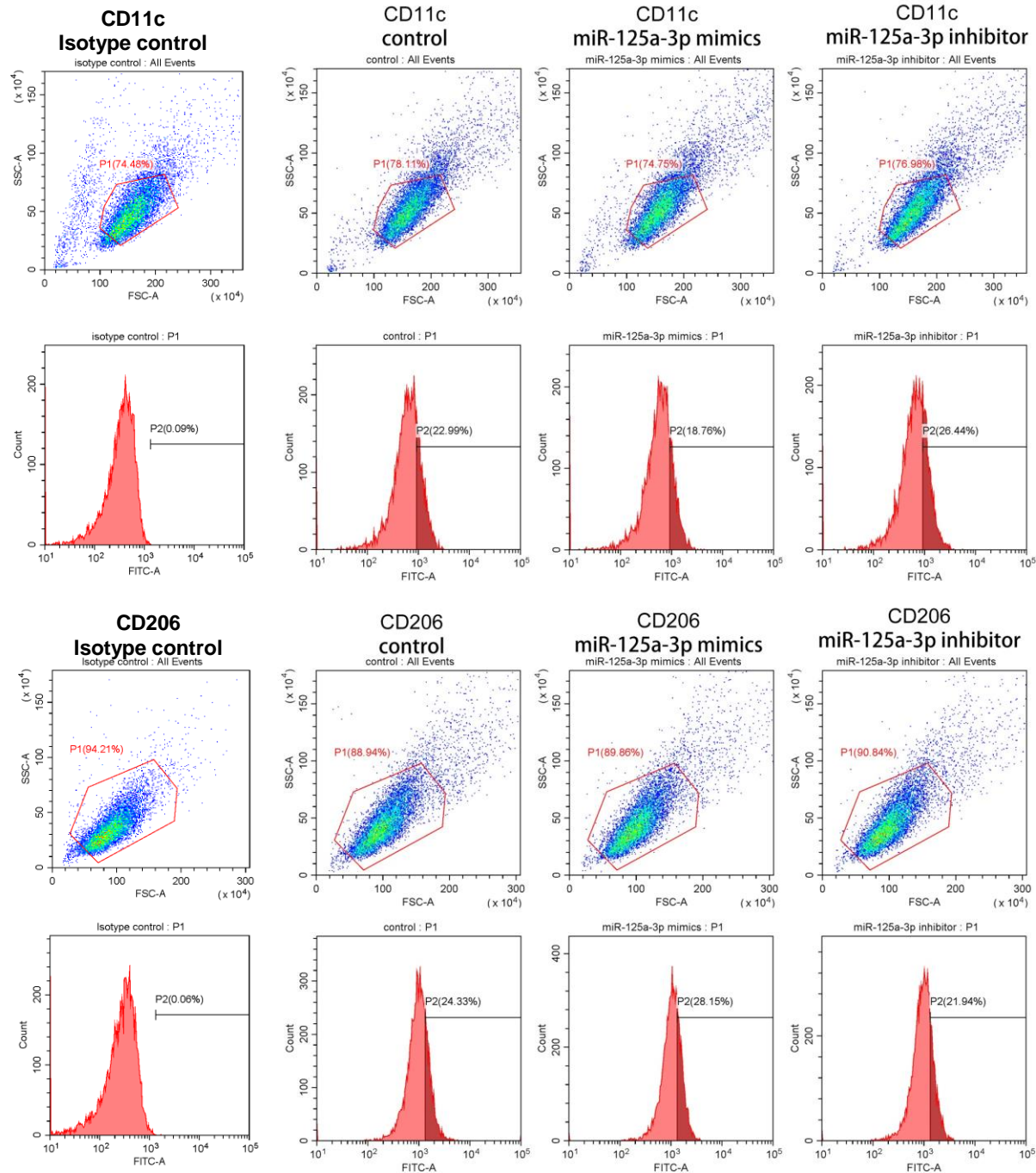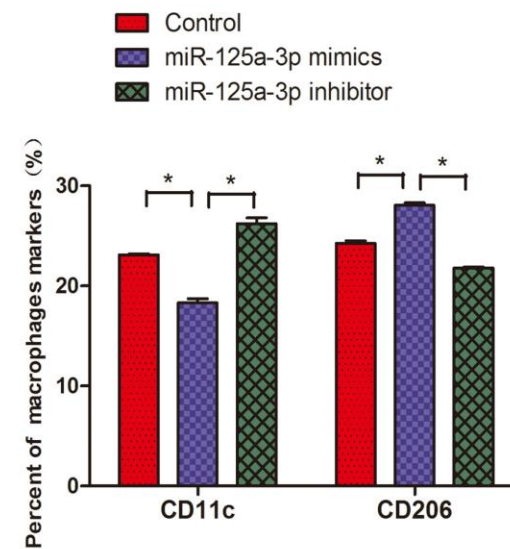

Supplement: Supplementary file 4 — Additional file 4: Figure S4. CD11c and CD206 in macrophages were suppressed and upregulated by miR-125a-3p mimics [file 13287_2020_2039_MOESM4_ESM.pdf]

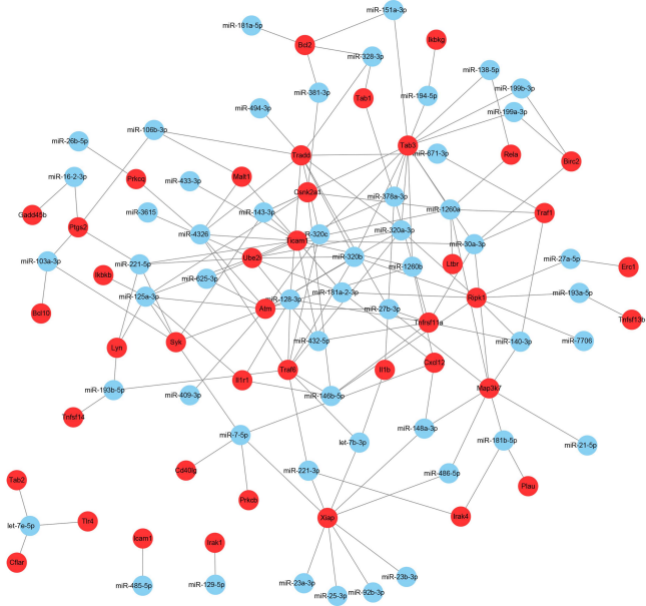

Supplement: Supplementary file 5 — Additional file 5: Figure S5. mRNA-microRNA interaction networks showed 41 genes in the NFκΒ pathway targeted by 53 microRNAs with differential expression [file 13287_2020_2039_MOESM5_ESM.pdf]

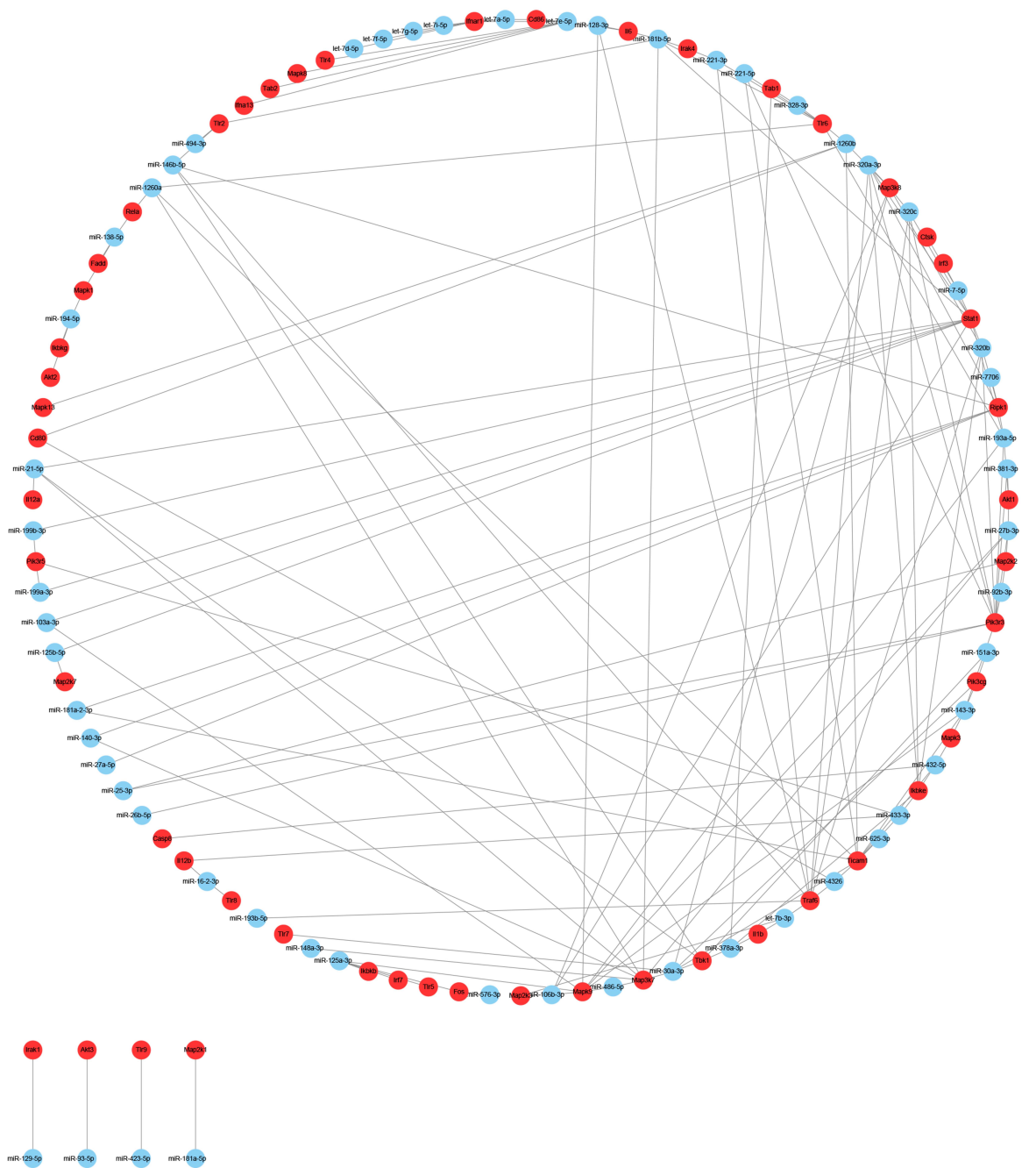

Supplement: Supplementary file 6 — Additional file 6: Figure S6. 51 in TLR signaling regulated by 56 microRNAs with differential expression [file 13287_2020_2039_MOESM6_ESM.pdf]
