# Supplementary material for: MicroRNA-enriched small extracellular vesicles possess odonto-immunomodulatory properties for modulating the immune response of macrophages and promoting odontogenesis
Source: Stem Cell Res Ther. 2020 Nov 30;11:517. doi: 10.1186/s13287-020-02039-1 (PMC7708107; doi:10.1186/s13287-020-02039-1)
Supplement: Supplementary file 2 — Additional file 2: Figure S2. Original non-edited images of Chemiluminescence intensity and the automatically generated bands for MyD88and β-tubulin. (A) DPSCs-sEV down-regulated the expression of MyD88 in macrophages. (B) miR-125a-3p down-regulated the expression of MyD88 in macrophages [file 13287_2020_2039_MOESM2_ESM.pdf]

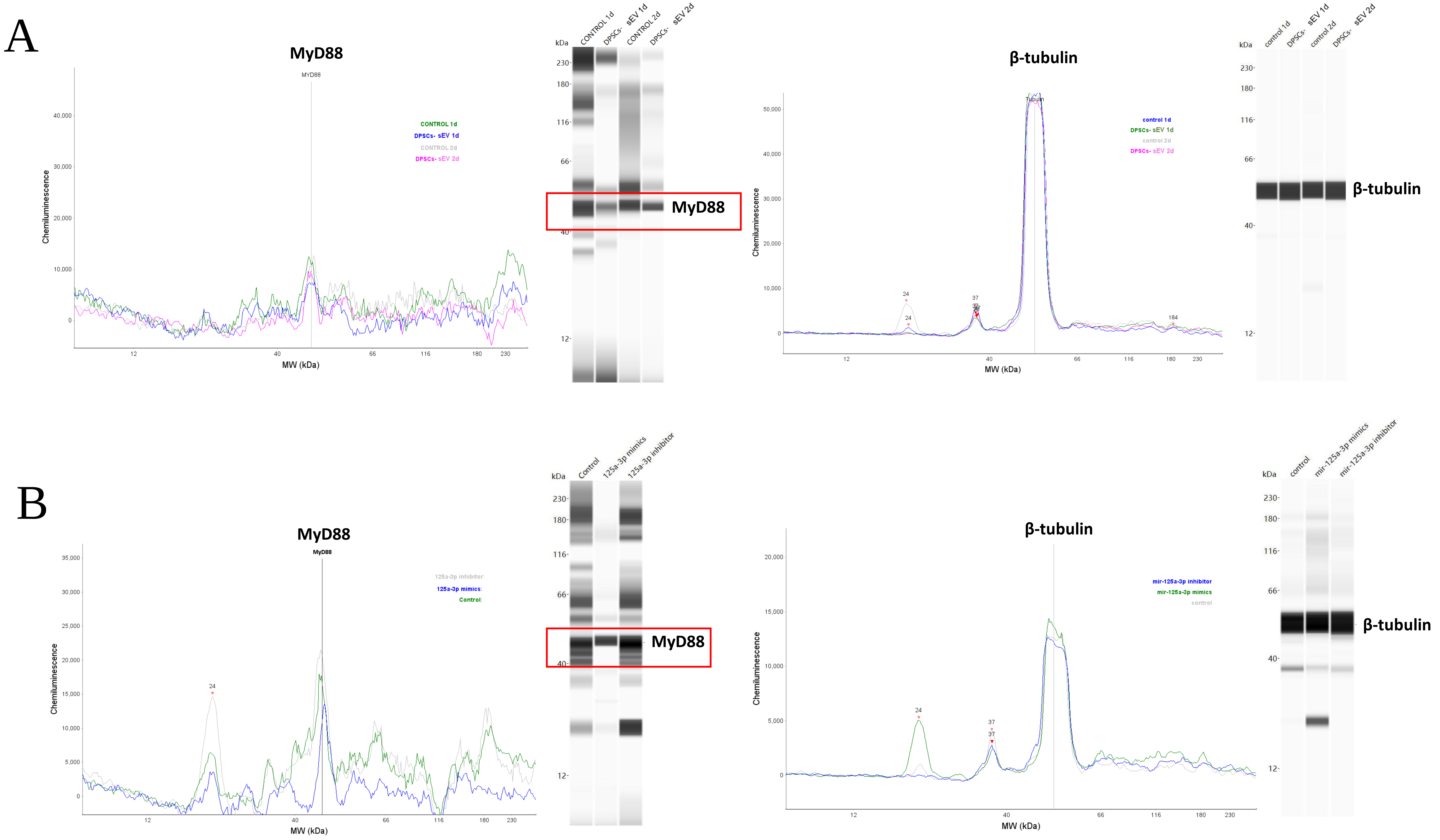

Figure S2. Original non-edited images of Chemiluminescence intensity and the automatically generated bands for MyD88 and β-tubulin. (A) DPSCs-sEV down-regulated the expression of MyD88 in macrophages. (B) miR-125a-3p down-regulated the expression of MyD88 in macrophages.
