# Supplementary material for: MicroRNA-enriched small extracellular vesicles possess odonto-immunomodulatory properties for modulating the immune response of macrophages and promoting odontogenesis
Source: Stem Cell Res Ther. 2020 Nov 30;11:517. doi: 10.1186/s13287-020-02039-1 (PMC7708107; doi:10.1186/s13287-020-02039-1)
Supplement: Supplementary file 7 — Additional file 7: Table S1. Primer pairs used in the qRT-PCR. [file 13287_2020_2039_MOESM7_ESM.docx]

Table S1. Primer pairs used in the qRT-PCR.

| ID | Sequence(5’- 3’) |
| --- | --- |
| β-actin.F | CATTGCTGACAGGATGCAGA |
| β-actin.R | CTGCTGGAAGGTGGACAGTGA |
| IL6.F | CTGCAAGAGACTTCCATCCAG |
| IL6.R | AGTGGTATAGACAGGTCTGTTGG |
| TNF-α.F | CCCACGTCGTAGCAAACC |
| TNF-α.R | GATAGCAAATCGGCTGACGG |
| IL-1β.F | TGATAACCTGCTGGTGTGTG |
| IL-1β.R | AGGCCACAGGTATTTTGTCG |
| IL10.F | TTGGGTTGCCAAGCCTTATC |
| IL10.R | TGATTTCTGGGCCATGCTTC |
| IL1ra.F | ACCCTACAGTCACCTAATCTCT |
| IL1ra.R | TGGATGCCCAAGAACACACTA |
| TGFβ1.F | GAGCCCGAAGCGGACTACTA |
| TGFβ1.R | TGGTTTTCTCATAGATGGCGTTG |
| TGFβ3.F | AAAGGGCTCTGGTAGTCCTG |
| TGFβ3.R | CCTAATGGCTTCCACCCTCT |
| IKBKB.F | GATCGCCTGTAGCAAAGTCC |
| IKBKB.R | GTCTTGCTCCTTCACAGTGTC |
| BMP2.F | TTGGACACCAGGTTAGTGAATC |
| BMP2.R | CCTGGGTTCTCCTCTAAATGG |
