# Supplementary material for: MicroRNA-enriched small extracellular vesicles possess odonto-immunomodulatory properties for modulating the immune response of macrophages and promoting odontogenesis
Source: Stem Cell Res Ther. 2020 Nov 30;11:517. doi: 10.1186/s13287-020-02039-1 (PMC7708107; doi:10.1186/s13287-020-02039-1)
Supplement: Supplementary file 8 — Additional file 8 Table S2. There were 81 microRNAs significantly changed in DPSCs-sEV, of which 54 increased and 27 decreased. [file 13287_2020_2039_MOESM8_ESM.docx]

Table S2. There were 81 microRNAs significantly changed in DPSCs-sEV, of which 54 increased and 27 decreased.

| miRNA_ID | up/down | fold change | Significance |
| --- | --- | --- | --- |
| hsa-miR-625-3p | up | 34 | ** |
| hsa-miR-423-5p | up | 23.6 | ** |
| hsa-miR-486-5p | up | 34.4 | ** |
| hsa-miR-148a-3p | up | 15.4 | ** |
| hsa-miR-193a-5p | up | 23.9 | ** |
| hsa-miR-92b-3p | up | 20.1 | ** |
| hsa-miR-4326 | up | 27.6 | ** |
| hsa-miR-129-5p | up | 36 | ** |
| hsa-miR-194-5p | up | 26.9 | ** |
| hsa-miR-485-5p | up | 21.5 | ** |
| hsa-miR-192-5p | up | 26.7 | ** |
| hsa-miR-409-3p | up | 13.3 | ** |
| hsa-miR-134-5p | up | 14 | ** |
| hsa-miR-140-3p | up | 2.3 | ** |
| hsa-miR-10a-5p | up | 26.1 | ** |
| hsa-miR-100-5p | up | 2.6 | ** |
| hsa-miR-23a-3p | up | 2.6 | ** |
| hsa-miR-218-5p | up | 2.1 | ** |
| hsa-miR-143-3p | up | 2.4 | ** |
| hsa-miR-543 | up | 9.7 | ** |
| hsa-let-7d-3p | up | 13.2 | ** |
| hsa-miR-432-5p | up | 18.1 | ** |
| hsa-miR-7706 | up | 24.1 | ** |
| hsa-miR-423-3p | up | 8.4 | ** |
| hsa-miR-191-5p | up | 2.6 | ** |
| hsa-miR-99b-5p | up | 2.6 | ** |
| hsa-miR-671-3p | up | 12.4 | ** |
| hsa-miR-320b | up | 12.1 | ** |
| hsa-let-7a-3p | up | 2.2 | ** |
| hsa-miR-193b-5p | up | 22.4 | ** |
| hsa-miR-146b-5p | up | 2.3 | ** |
| hsa-miR-99a-5p | up | 3.1 | ** |
| hsa-miR-494-3p | up | 2.2 | ** |
| hsa-miR-138-5p | up | 2.2 | ** |
| hsa-miR-328-3p | up | 8.2 | ** |
| hsa-miR-151a-3p | up | 7.5 | ** |
| hsa-miR-3615 | up | 10.7 | ** |
| hsa-miR-30a-3p | up | 3.2 | ** |
| hsa-miR-30d-5p | up | 3.1 | ** |
| hsa-miR-378a-3p | up | 8.1 | ** |
| hsa-miR-433-3p | up | 9.8 | ** |
| hsa-let-7b-3p | up | 2.8 | * |
| hsa-miR-381-3p | up | 3.7 | * |
| hsa-miR-339-5p | up | 2.6 | * |
| hsa-miR-576-3p | up | 15.1 | * |
| hsa-miR-16-2-3p | up | 2.6 | * |
| hsa-miR-106b-3p | up | 2.7 | * |
| hsa-miR-1307-3p | up | 3.4 | * |
| hsa-miR-320a-3p | up | 7.3 | * |
| hsa-miR-320c | up | 12.8 | * |
| hsa-miR-27a-5p | up | 7.9 | * |
| hsa-miR-128-3p | up | 6.8 | * |
| hsa-miR-25-3p | up | 6.7 | * |
| hsa-miR-125a-3p | up | 12.5 | * |
| hsa-miR-26a-5p | down | 5.1 | ** |
| hsa-miR-221-3p | down | 3.4 | ** |
| hsa-let-7i-5p | down | 4.1 | ** |
| hsa-miR-199b-3p | down | 5.4 | ** |
| hsa-miR-199a-3p | down | 5.4 | ** |
| hsa-miR-26b-5p | down | 11.3 | ** |
| hsa-miR-21-5p | down | 3.3 | ** |
| hsa-let-7f-5p | down | 10.6 | ** |
| hsa-miR-181a-5p | down | 4.3 | ** |
| hsa-let-7g-5p | down | 3 | ** |
| hsa-let-7a-5p | down | 19 | ** |
| hsa-miR-199a-5p | down | 3 | ** |
| hsa-let-7d-5p | down | 4.1 | ** |
| hsa-miR-23b-3p | down | 2.7 | ** |
| hsa-miR-125b-5p | down | 2.4 | ** |
| hsa-let-7e-5p | down | 10.9 | ** |
| hsa-miR-103a-3p | down | 7.1 | ** |
| hsa-miR-27b-3p | down | 2.6 | ** |
| hsa-miR-221-5p | down | 10.2 | ** |
| hsa-miR-181b-5p | down | 2.3 | ** |
| hsa-miR-379-5p | down | 2.4 | ** |
| hsa-miR-7-5p | down | 2.9 | ** |
| hsa-miR-93-5p | down | 2.3 | ** |
| hsa-miR-186-5p | down | 2.6 | ** |
| hsa-miR-181a-2-3p | down | 2.2 | ** |
| hsa-miR-1260b | down | 3 | ** |
| hsa-miR-1260a | down | 3 | ** |

*, *p*<0.05; **, *p*<0.01
